# An Uncommon Presentation of Spontaneous Rectus Sheath Hematoma with Acute Kidney Injury due to Obstructive Uropathy and Prerenal Azotemia

**DOI:** 10.1155/2014/164245

**Published:** 2014-07-13

**Authors:** Eleni Paschou, Eleni Gavriilaki, Asterios Kalaitzoglou, Maria Mourounoglou, Nikolaos Sabanis

**Affiliations:** ^1^Department of Family Medicine, General Hospital of Pella, 58200 Edessa, Greece; ^2^Medical School, Aristotle University of Thessaloniki, 54124 Thessaloniki, Greece; ^3^Department of General Surgery, General Hospital of Pella, 58200 Edessa, Greece; ^4^Nephrological Department, General Hospital of Pella, 58200 Edessa, Greece

## Abstract

Rectus Sheath Hematoma (RSH) represents an unusual entity which is characterized by acute abdominal pain and tender palpable abdominal mass usually, among elderly patients receiving anticoagulant therapy. We report the case of an 81-year-old woman admitted to our department due to acute abdominal pain and oligoanuria. The patient had recently been hospitalized due to acute myocardial infarction (AMI) and atrial fibrillation (AF) and received both anticoagulant and antiplatelet therapies. The radiological assessments revealed an extended Rectus Sheath Hematoma and bilateral hydronephrosis. Treatment of the hematoma required cessation of anticoagulants and antiplatelet agents, immobilization, blood and fresh frozen plasma transfusion, and administration of vasopressors. The patient recovered gradually and was discharged home fifteen (15) days later.

## 1. Introduction

During the last years, anticoagulant and antiplatelet agents have been extensively used, as a treatment or prophylaxis of several conditions in increased thrombotic risk, such as venous thromboembolism, pulmonary embolism, acute coronary syndromes, atrial fibrillation and stroke. However, the benefits do not always outweigh the risks of antithrombotic therapy, since a number of adverse events have been reported. Rectus Sheath Hematoma (RSH) represents an uncommon complication of anticoagulant therapy that can be misdiagnosed because it mimics other causes of abdominal pain.

Herein, we report an interesting case of an elderly patient with abdominal pain caused by RSH who received conservative treatment in time, avoiding further complications.

## 2. Case Report

**Figure 1:**
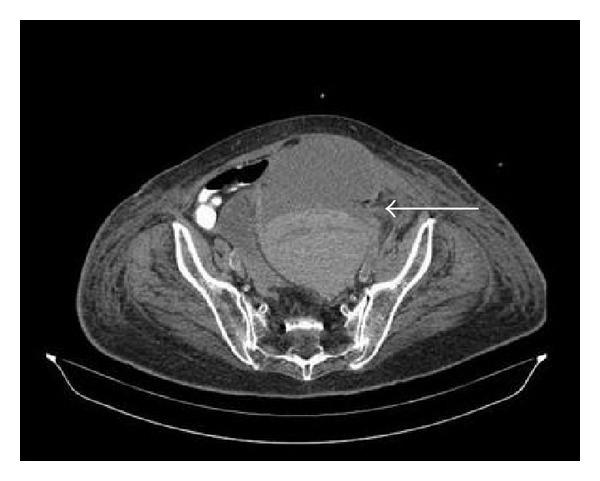
Rectus Sheath Hematoma**: **“hematocrit formation” point.

**Figure 2:**
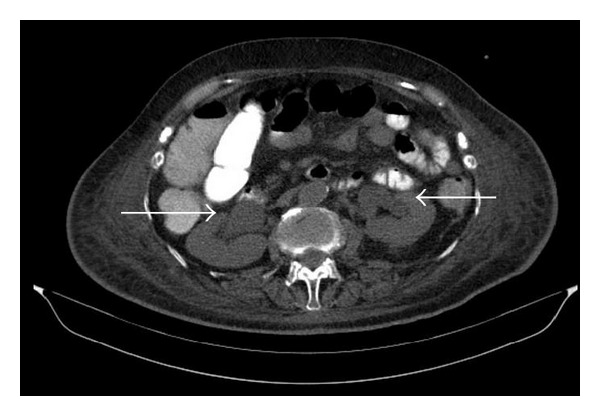
Rectus Sheath Hematoma**: **distension of renal pelvic system bilaterally.

An 81-year-old Caucasian female presented to our emergency department with acute abdominal pain and oligoanuria (urinary output 100 mL/8 h). Her medical history included a recent hospitalization due to acute myocardial infarction (AMI) and atrial fibrillation (AF), treated with amiodarone intravenous infusion (amiodarone 300 mg bolus iv) and anticoagulant therapy with acenocoumarol and dual antiplatelet treatment (aspirin 100 mg per day; clopidogrel 75 mg per day). During the first hospitalization, she developed amiodarone-induced hepatotoxicity. Therefore, the treatment with acenocoumarol was interrupted and replaced with low molecular weight heparin (LMWH) (nadroparin 5700 antiXA/0.6 mL twice daily) while antiplatelet treatment continued. Eight (8) days after she was discharged home, she presented in the emergency department hemodynamically unstable (BP: 80/60 mmHg, HR: 115 bpm) with tachypnea and low grade fever. Physical examination demonstrated a painful mass extending to the lower abdomen and upper ecchymosis. Routine laboratory examinations revealed anemia, acute renal failure, hypocalcaemia, and coagulation disturbances (Table 1-Day 1). Urinalysis showed acute tubular necrosis (Specific Gravity 1027, pH 6, WBCs 0-1, RBCs 0-1, and browncast cylindroids). Both abdominal ultrasonography and abdominopelvic CT scan demonstrated an hematoma at lower abdomen, on the left rectus abdominis muscle extending to the pelvis (dimensions 11.3 × 14.6 × 10.5 cm), presented with the “hematocrit formation” point (Figure 1). Furthermore, bilateral hydronephrosis was observed due to hematoma's invasive traits. Central venous pressure (CVP) was 3 mmHg and intra-abdominal pressure (IAP), using the intravesicular method, was 21 mmHg (abdominal compartment syndrome (ACS)). Acute kidney injury correlated with acute tubular necrosis and postrenal obstructive uropathy (Figure 2).

Anticoagulant and antiplatelet agents were ceased. On day 1, in accordance with CVP, intravenous fluids (0.9% sodium chloride solution) were infused in order to restore intravascular volume. The patient received two packed red blood cells transfusions, one fresh frozen plasma transfusion and calcium gluconate (10 mL calcium gluconate 10%/8 h) because of the blood clotting mechanism's disturbances. On day 2, despite normal ranges of CVP, the patient was hemodynamically unstable and was treated with vasopressors (dopamine 10 *μ*g/kg/min). The above measures increased blood pressure to 120 mmHg and the urinary output improved (400 mL/8 h). The patient recovered gradually after fifteen (15) days of bed rest, without any complications, and was discharged hemodynamically stable (BP: 115/80) with normal IAP (7 mmHg), renal and liver function (Table 1-Day 15), while she had received four packed red blood cells and six fresh frozen plasma transfusions in total.

## 3. Discussion

RSH is usually a self-limiting entity that potentially can lead to severe complications. Obstructive uropathy [1] and abdominal compartment syndrome [2] are uncommon complications even though RSH is involved with other rare entities such as hemoperitoneum [3], gross hematuria [4], rectus abdominis myonecrosis [5], ileocecal perforation [6], and small bowel infraction [7].

The mortality rate has been reported at 4% in general population and up to 25% in patients under anticoagulant therapy. It is more frequent in female and elderly patients, mainly because of their decreased muscle mass [8].

RSH is a rare cause of acute abdominal pain presenting with ecchymosis and abdominal wall mass due to rupture of epigastric vessels or arteries. It occurs usually unilateral, although some rare cases of bilateral hematomas have been reported, as complications of kidney transplantation [9] and alcohol liver disease [10]. Symptoms following the appearance of RSH are mainly nonspecific and include fever, hypovolaemia, nausea, vomiting, and diarrhea. Recognition of clinical signs such as Cullen, Grey-Turner, Carnett (tenderness remains the same or increases with head raising) [11, 12], and Fothergill's sign (the abdominal mass in RSH does not cross the midline and, in contrast to an intraperitoneal mass, it remains conspicuous on tensing the abdominal wall musculature by head or leg raising) [13] may be beneficial for diagnostic approach.

The main risk factors for RSH are anticoagulant therapies, coagulation disorders, previous surgical operations [14], abdominal trauma, increased intra-abdominal pressure (cough, sneezing, strenuous exercise [15, 16], pregnancy [17], and constipation), cardiovascular diseases, and myopathies [18]. Other causes have been also described in case reports, such as acupuncture [19], subcutaneous injection, foley catheterization [20], endometriosis of rectus abdominis, transvaginal follicle aspiration during* in vitro* fertilisation [21], HCV-related mixed cryoglobulinemia [22], lymphoproliferative disease after renal transplantation [23], and tetanus [24].

The role of ultrasonography and computed tomography is crucial, although computed tomography appears to be the most accurate way of confirming the diagnosis [25].

According to Berná et al. [26] and Osinbowale and Bartholomew [13] RSH can be classified into three categories that can lead to appropriate therapeutic strategies (Table 2).

There have been reported only few cases of RSH complicated with acute kidney injury. The causes in these cases seem to be prerenal, intrarenal, or postrenal. Our patient appeared with both prerenal and postrenal causes. Patient's hemodynamic instability caused prolonged renal ischemia which led to acute tubular necrosis while the bilateral obstructive uropathy caused significant raise of intratubular pressure. As a result of obstructive uropathy, renal blood flow decreased further leading to acute kidney injury.

The pathophysiological mechanisms of blood clotting disturbances in this case are complicated and involve uremia, accumulation of LMWH, and anticoagulant therapy. It is well known that uremia in patients with renal insufficiency leads to qualitative platelet abnormalities, mainly caused by A2 thromboxane reduced production due to abnormal platelet arachidonic acid metabolism [27]. In these patients, heparin levels should be reduced, especially when creatinine clearance is less than 40 mL/min [28], in order to prevent complications. The remarkable points in our case report were that the patient had also amiodarone-induced hepatotoxicity and hypocalcaemia causing further disturbances of blood clotting mechanism [29].

RSH management is mainly supportive, including immobilization, cessation of anticoagulation therapy, and transfusions. Angioembolization may be necessary [30] especially for RSHs related to LMWH [31] and surgical intervention should be reserved for cases with hemodynamic instability which resist in conventional treatment [32].

## 4. Conclusions

RSH should be in mind of physicians during differential diagnosis of acute abdominal pain, especially in elderly patients receiving anticoagulants. The causal nature remains unclear since the underlying pathophysiological pathways are complicated. Early recognition can be of great importance for patients' recovery, preventing from severe complications. Management is usually supportive although surgical intervention in some patients should be considered.

## Figures and Tables

**Table 1:** Routine laboratory examination.

	Day 1	Day 2	Day 3	Day 7	Day 15
WBC count (×10^3^/*μ*L)	12,36	13,57	13,12	9,78	7,65
Hemoglobulin (g/dL)	7,9	8,6	8,8	11	11,5
Hematocrit (%)	24,2	27,7	29,8	33,5	36,2
Platelet count (×10^3^/*μ*L)	145	157	159	162	154
Serum creatinine (mg/dL)	2,73	2,69	2,14	1,56	1,12
Urea (mg/dL)	140	114	98	60	43
SGOT (mg/dL)	226	234	217	85	36
SGPT (mg/dL)	432	392	366	109	40
Serum calcium (mg/dL)∗	6,7	6,9	7,1	7,6	8,2
Activated partial thromboplastin time (aPTT) (sec)	38,7	36,7	32	29,9	27,9
Fibrinogen (g/L)	1,53	1,66	2,01	2,6	2,9
International normalized ratio (INR)	1,84	1,67	1,34	1,27	1,1

*corrected to albumin.

**Table 2:** Berna and Osinbowale RSH classification. Computed tomography severity grades and suggested management strategy, modified and reprinted with permission from Osinbowale and Bartholomew [13].

Grade	Anatomic extension	Symptoms	Management
I	Intramuscular, unilateral; does not dissect along fascial planes.	Mild to moderate pain. No drop in hemoglobin.	Conservative; usually outpatient follow-up only.

II	Bilateral; some dissection between the muscle and transversalis fascia; no extension into the prevesical space.	Minor drop in hemoglobin.	Observation, short hospital stay. May need transfusion.

III	Bilateral, large; dissects between the transversalis fascia and muscle into the peritoneum and prevesical space.	Significant drop in hemoglobin and hemodynamic instability.	Reversal of anticoagulants and blood transfusion. Angiographic interventions may be needed.
